# Design of a Phase 1, First‐in‐human, Randomized, Double‐blind, Placebo‐controlled, Single and Multiple Ascending Dose Study of a Novel Orally Administered TREM2 Agonist (VG‐3927) in Healthy Volunteers

**DOI:** 10.1002/alz.089977

**Published:** 2025-01-09

**Authors:** Raj Rajagovindan, Francois Gaudreault, Ryan O’Mara, Evan A. Thackaberry, Jessica Stromme, David L. Gray, Andreas Meier

**Affiliations:** ^1^ Vigil Neuroscience, Inc, Watertown, MA USA

## Abstract

**Background:**

VG‐3927 is a highly potent, selective, brain penetrant, oral small molecule TREM2 agonist that is currently under development for the treatment of Alzheimer’s disease (AD). TREM2, a receptor expressed on microglia in the brain is critical to microglial function in health and in disease. Among microglia‐associated AD risk genes, partial loss‐of‐function variants of TREM2 confer 2‐3 fold increase in risk for developing AD, motivating efforts to identify pharmacological agonists targeting TREM2 as a therapeutic option. VG‐3927 is designed to act as a molecular glue that potentiates the TREM2 signaling response to natural damage ligands and is hypothesized to promote microglial function and health, and tissue repair mechanisms regulated by microglia thereby slowing disease progression in AD.

**Methods:**

The Phase 1 randomized, double‐blind, placebo‐controlled study will evaluate the safety, tolerability, pharmacokinetics, and pharmacodynamics after single‐ (SAD) and multiple‐ascending dose (MAD) of orally administered VG‐3927 in healthy volunteers. The study will enroll healthy adults aged 18 to 55 years and include SAD and MAD cohorts that are planned to be tested sequentially in a dose‐escalating manner. Eight participants will be randomized in a 6:2 allocation ratio to VG‐3927 or placebo into each of the planned cohorts. The treatment period for the SAD and the MAD portions will be 1 day and 14 days respectively with safety follow‐up through 28 days after the last dose for both portions. Study assessments to investigate safety and tolerability will include physical exams, neurological exams, vitals, ECG, clinical laboratory, C‐SSRS and AE reporting in all cohorts. Blood samples will be collected in all cohorts to characterize the pharmacokinetics and CSF samples will be collected in selected cohorts to investigate biomarkers of target engagement and downstream pharmacodynamic activity.

**Results:**

The study dosed its first participant in October 2023 and is currently ongoing. The design of the Phase 1 study will be presented.

**Conclusions:**

The ongoing Phase 1 study will assess the safety, tolerability, pharmacokinetics, and pharmacodynamics of VG‐3927 in healthy volunteers and is expected to inform subsequent development in AD.

